# Supplementary material for: Next-Generation Antisense Oligonucleotide of TGF-β2 Enhances T Cell-Mediated Anticancer Efficacy of Anti-PD-1 Therapy in a Humanized Mouse Model of Immune-Excluded Melanoma
Source: Cancers (Basel). 2022 Oct 25;14(21):5220. doi: 10.3390/cancers14215220 (PMC9654371; doi:10.3390/cancers14215220)
Supplement: Supplementary file 1 [file cancers-14-05220-s001.zip › cancers-1936511-supplementary.pdf]

Supplementary

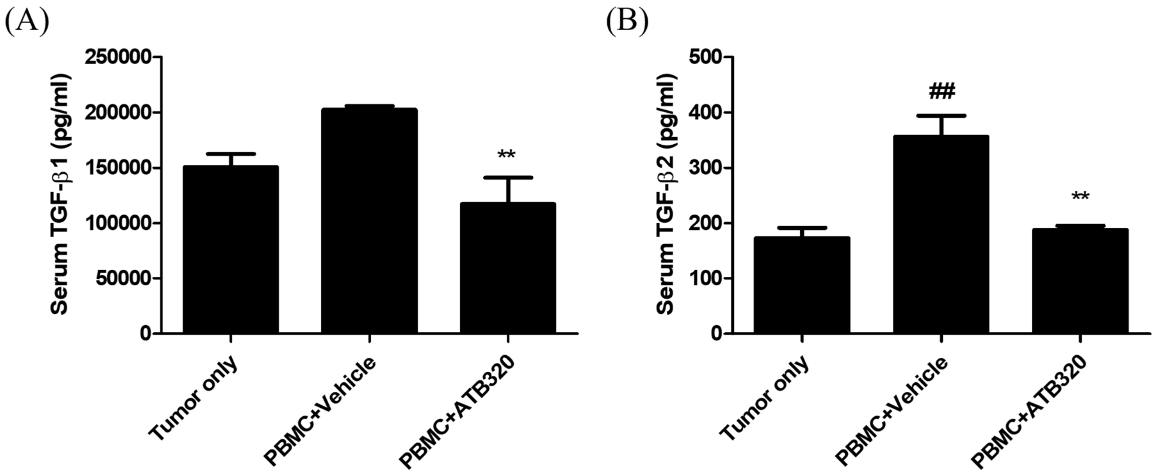

Figure S1. ***Effects of ATB320 on changes in serum TGF-βs levels.*** Levels of TGF-β1 **and** TGF-β2 in the blood serum of hu-PBL NSG-B2m mice were detected by the Luminex assay on day 23. Data showing the concentration of (A) TGF-β1 **and (B)** TGF-β2 **in serum. The results are expressed as mean±S.E.M. obtained from six mice per group.** ## $p < 0.01$ vs. Tumor only group; ** $p < 0.01$ vs. PBMC+Vehicle group (Dunnet's test).
